# Factors associated with selection of targeted therapy in patients with rheumatoid arthritis

**DOI:** 10.1371/journal.pone.0280234

**Published:** 2023-01-10

**Authors:** Yeo-Jin Song, Soo-Kyung Cho, Hyoungyoung Kim, Hye Won Kim, Eunwoo Nam, Chan-Bum Choi, Tae-Hwan Kim, Jae-Bum Jun, Sang-Cheol Bae, Dae Hyun Yoo, Yoon Kyoung Sung

**Affiliations:** 1 Department of Rheumatology, Hanyang University Hospital for Rheumatic Diseases, Seoul, Republic of Korea; 2 Hanyang University Institute for Rheumatology Research, Seoul, Republic of Korea; Qatar University, QATAR

## Abstract

**Objective:**

Deciding which drug to choose for targeted therapy is an important step in sequential treatment for rheumatoid arthritis (RA). This study aimed to identify factors for selecting Janus kinase inhibitors (JAKis) rather than biologic disease-modifying antirheumatic drugs (bDMARDs) in patients with RA in real-world practice.

**Methods:**

We selected RA patients starting JAKis or bDMARDs from single-center prospective cohorts in Korea. Patients were divided into JAKi, tumor necrosis factor (TNF) inhibitor, and non-TNF inhibitor groups. We performed multinomial logistic regression analyses to identify factors associated with selecting JAKis.

**Results:**

145, 205, and 89 patients were included in the JAKi, TNF inhibitor, and non-TNF inhibitor groups. In multinomial regression analysis, the JAKi group was older than the TNF inhibitor group (OR 1.03, 95% confidence interval [CI] 1.01–1.05) but younger than the non-TNF inhibitor group (OR 0.97, CI 0.95–1.00). The JAKi group was less likely to have chronic pulmonary diseases compared with the TNF inhibitor group (OR 0.07, CI 0.01–0.56) or the non-TNF inhibitor group (OR 0.06, CI 0.01–0.50). Higher disease activity assessed by physician (OR 1.80, CI 1.51–2.38) and previous tacrolimus use (OR 2.05, CI 1.20–3.51) were factors suggesting selection of JAKis than TNF inhibitors.

**Conclusion:**

Age, pulmonary comorbidities, previous tacrolimus use, and high disease activity assessed by physician were factors influencing the selection of JAKis for RA patients in Korea.

## Introduction

The successful introduction of targeted therapy for rheumatoid arthritis (RA) was made possible by improved understanding of the pathogenesis of RA [1], and has increased the attainment of clinical remission or low disease activity in RA [2]. Targeted therapy for RA is classified into use of tumor necrosis factor (TNF) inhibitor, non-TNF inhibitor, and Janus kinase inhibitor (JAKi), the latter of which is the most recently released. Many clinical studies have verified the efficacy and safety of these targeted therapies, followed by studies using real world data for determining long-term safety [3–7]. The drugs used for targeted therapy have different mode of action, but there are no clinically important differences in efficacy [8]. Therefore, JAKis, TNF inhibitors, and non-TNF inhibitors are recommended for RA patients who show inadequate response (IR) to conventional synthetic disease-modifying antirheumatic drugs (csDMARDs) through a shared decision-making process between patients and physicians [9,10].

TNF inhibitor was the first biologic agent used for treatment of RA, and adalimumab, etanercept, golimumab, and infliximab are currently approved for use in Korea [11]. These drugs share efficacy and safety profiles, as well as concerns about hematologic malignancy and opportunistic infections such as tuberculosis [12]. Abatacept and tocilizumab are non-TNF inhibitors available for RA, and both have been shown to have similar efficacy as TNF inhibitors [3,13]. However, the efficacies of non-TNF inhibitors and TNF inhibitors as monotherapy were different: abatacept and tocilizumab have been used as monotherapies with similar efficacy as in combination with methotrexate (MTX), while TNF inhibitors have been recommended to be used in combination with MTX [14]. There were no significant differences in overall safety compared to TNF inhibitor [12,15], but abatacept was reported to show a lower risk of hospitalized infection than TNF inhibitor [16]. In addition, abatacept is considered safe for patients with pulmonary comorbidities such as interstitial lung disease (ILD) and chronic obstructive pulmonary disease (COPD) [17,18]. Though rituximab is another biologic DMARD (bDMARD) available for csDMARD-IR patients with RA, it is not approved as second-line therapy in Korea.

Recently, JAKis have been developed for RA treatment: tofacitinib was the first JAKi to be released and approved for RA treatment (in 2015), and baricitinib and upadacitinib are also currently available in Korea [3,19]. JAKis are low-molecular-weight compounds and can be conveniently administered orally, unlike other therapies that require injections. Hence, a great advantage of JAKis is that they are not associated with injection site reactions caused by subcutaneous injections of bDMARDs [20]. However, safety issues such as increased risk of herpes zoster and thromboembolism are worrisome for JAKi users [21,22].

New guidelines suggested sequential treatment according to a treat-to-target (T2T) strategy when using targeted therapy, and have led to significant changes in the paradigm of RA treatment [9]. Deciding which drug to choose as a second line therapy between bDMARD and JAKi is the most important step for sequential treatment. Availability is a main factor to consider first, but selection of targeted therapy can be influenced by both patient- and physician-related factors [20,23]. For example, route of administration could be an important factor in determining the patient’s preference due to the fears of injection and of side effects at injection sites [24,25]. On the other hand, physician factors such as changes in guidelines and personal experiences with drugs might influence the selection of targeted therapy [26].

In this study, we aimed to identify factors influencing the selection of JAKi as a first targeted therapy in patients with RA who were refractory to csDMARDs in real-world practice.

## Methods

### Data source and study population

This study used the baseline data of two prospective cohorts at an academic referral hospital in Korea. The Hanyang University Medical Center Arthritis Network-BIOlogics Registry for RA (HUMAN-BIORRA) cohort included RA patients receiving bDMARDs, and the HUMAN-Small Molecule Inhibitor Registry for RA (SMIRRA) cohort included patients receiving JAKis. The HUMAN-BIORRA and the HUMAN-SMIRRA were established in 2011 and 2016, respectively. To be enrolled in the cohorts, patients with RA had to satisfy the 1987 American College of Rheumatology (ACR) classification criteria for RA or the 2010 ACR/European Alliance of Associations for Rheumatology (EULAR) classification criteria. All patients with RA who started targeted therapy in our institution were admissible to the cohorts, but those who refused to give informed consent were excluded. The aim of the registries was to compare the effectiveness and safety of the targeted therapies.

Patients enrolled in these cohorts received physical examinations and interviews on the day of registration to collect enrollment and follow-up data. The data included demographic features such as age, sex, and body mass index (BMI), and clinical information such as comorbidities, medication history, and laboratory results. In addition, information related to RA was investigated including disease duration, disease activity and patient-reported outcomes. Information was collected from the patients every 6 months.

In Korea, a JAKi was first approved as a third line therapy for RA patients with bDMARD-IR in early 2015; in 2017 its indication was extended as a second line therapy for csDMARD-IR patients with RA by Korea Health Insurance Review and Assessment in accordance with the notice of the Ministry of Health and Welfare [27]. Hence, given the availability of drugs, we selected patients who started targeted therapy between March 2017 and August 2020, and divided them into three groups in accordance with the type of targeted therapy they received: JAKi, TNF inhibitor and non-TNF inhibitor groups.

### Factors associated with selecting JAKi

We considered all factors likely to affect the choice of JAKis. Based on the variables investigated in the cohorts, we analyzed factors that induced physician and patients to choose JAKi instead of TNF inhibitor or non-TNF inhibitor. These variables included the demographic and clinical information described above. Laboratory findings included rheumatoid factor (RF), anti-citrullinated protein antibody (ACPA), erythrocyte sedimentation rate (ESR), and C-reactive protein (CRP). In addition, medication histories of csDMARDs, oral glucocorticoids, and non-steroidal anti-inflammatory drugs (NSAIDs) were included. The patient-related factors were demographic and clinical information, including duration of RA, laboratory findings, Disease Activity Score of 28 joints (DAS28)-ESR, Health Assessment Questionnaire-Disability Index (HAQ-DI), EuroQoL-5 dimension (EQ-5D), and medication history. The HAQ-DI is the mean of the scores for eight categories assessing: arising, walking, dressing, hygiene, eating, reaching, gripping, and performing specific activities, each rated on a scale from 0 (without any difficulty) to 3 (unable to do) [28]. The EQ-5D assesses mobility, self-care, usual activities, pain/discomfort, and anxiety/depression, with an index score between 0 (death) and 1 (perfect health) [28]. Disease activity assessed by physician (Physician’s Global Assessment, PGA) was considered a physician-related factor. Differences in physician’s experience may influence the factors considered in drug selection. After discussing the subjective interpretation of these objective factors, four rheumatologists agreed to include those factors that they considered valuable as surrogate markers.

### Statistical analyses

We compared the demographic and clinical characteristics of patients from the three groups. The baseline characteristics are presented as means with standard deviations or frequencies with percentages. The chi-square test was used for categorical variables, and the Kruskal-Wallis test was used for continuous variables.

To identify factors inducing the initiation of JAKi rather than bDMARDs, we performed binary logistic regression analysis. Variables significantly different in the univariable analysis and of interest were included in the multivariable analysis. Multinomial regression analysis was also performed for comparison between JAKi and TNF inhibitor or non-TNF inhibitor.

All analyses were performed using SAS 9.4 (SAS Institute, Cary, NC, USA), and P values <0.05 were considered statistically significant.

### Ethical considerations

This study used data from two cohorts which was previously approved by the Institutional Review Board (IRB) of Hanyang University Hospital (IRB No. HYUH 2011-05-008, HYUH 2016-08-037, HYUH 2018-12-024). The study was conducted in accordance with the declaration of Helsinki and written informed consent was obtained from all patients enrolled in the cohorts. Data from each cohort did not include patients’ personal information, and this retrospective analysis was separately approved by IRB in Hanyang University Hospital (IRB No. HYUH 2021-03-015).

## Results

### Baseline characteristics of RA patients

**Fig 1:**
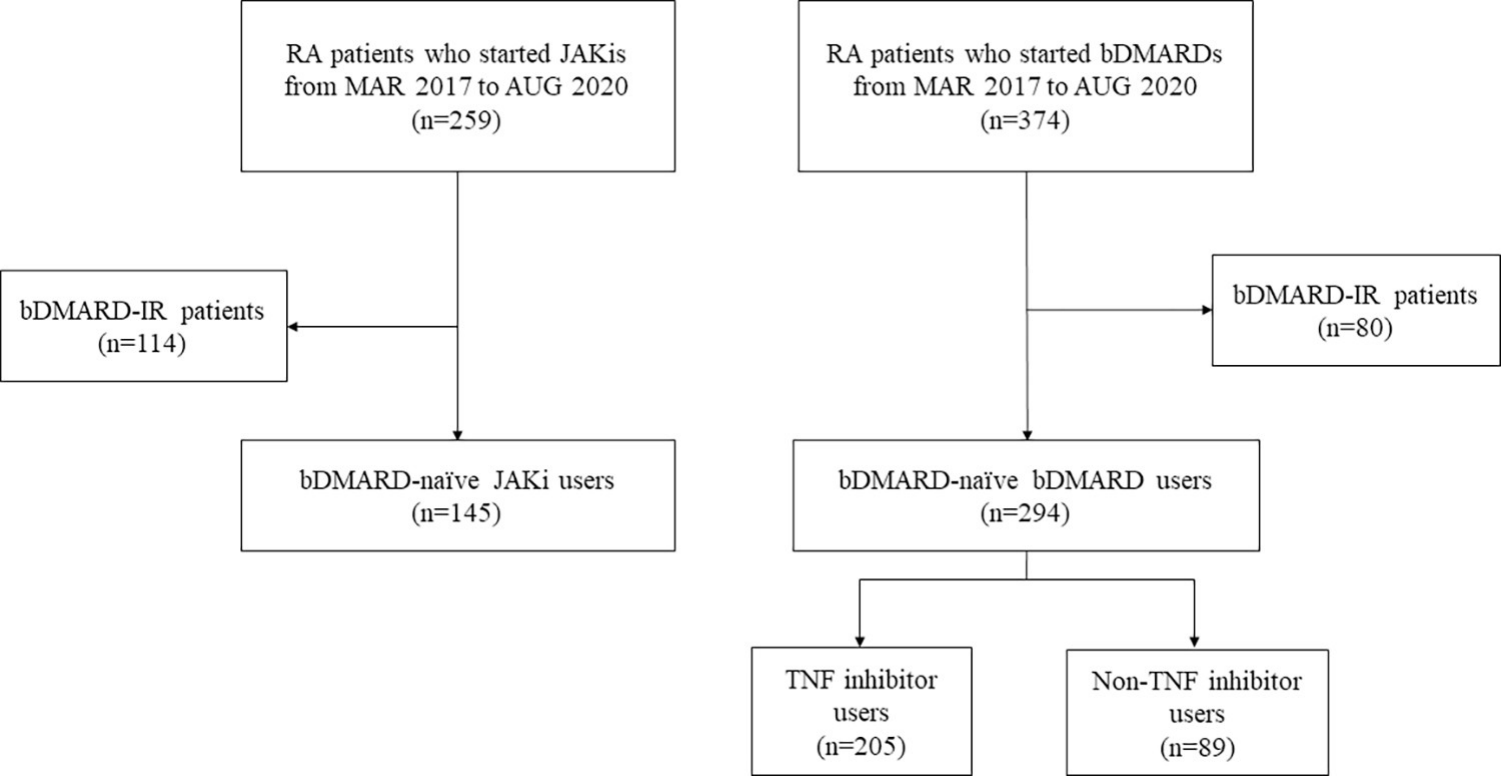
Flow of patient selection. RA, rheumatoid arthritis; JAKi, Janus kinase inhibitor; bDMARD, biological disease-modifying antirheumatic drugs; IR, inadequate response; TNF, tumor necrosis factor.

There were 633 patients who started JAKis or bDMARDs from March 2017 to August 2020 (Fig 1). After excluding bDMARD-IR patients, a total of 439 patients who had been naïve to targeted therapy were included in this study. Among them, 145 patients started JAKi, and 205 and 89 patients started TNF inhibitors and non-TNF inhibitors, respectively. The mean age of the study population was 53.1 years, and 88.4% were female (Table 1). Patients in the non-TNF inhibitor group tended to be older than the other groups. The duration of RA in total participants was 7.6 ± 7.8 years and was longest in the JAKi group. Patients with latent tuberculosis infection (LTBI) comprised 18.7% of the population, and those with chronic pulmonary disease comprised 7.1%. Patients with chronic pulmonary disease were less likely to be in the JAKi group, while there were no significant differences in the prevalence of LTBI between groups. Patients with ILD made up 6.4% of the population and were relatively common in the non-TNF inhibitor group. The disease activity of RA tended to be higher in the JAKi group, but ESR was lower in the JAKi group. Patients who previously used MTX and leflunomide were most common in the TNF inhibitor group, and those who previously used tacrolimus were most common in the JAKi group.

**Table 1:** Baseline characteristics of RA patients who initiated targeted therapy.

Variables	Total (n = 439)	JAKi (n = 145)	TNF inhibitor (n = 205)	Non-TNF inhibitor (n = 89)	*P*
Sex, female	388 (88.4)	122 (84.1)	187 (91.2)	79 (88.8)	0.125
Age, years	53.1 ± 14.1	55.2 ± 12.5	49.0 ± 14.1	58.9 ± 13.8	<0.001
Duration of RA, years	7.6 ± 7.8	9.4 ± 8.9	6.5 ± 6.8	7.3 ± 7.8	0.005
Body mass index, kg/m^2^ (n = 437,143,205,89)	22.5 ± 3.6	22.6 ± 3.6	22.4 ± 3.7	22.6 ± 3.1	0.531
**Comorbidities**					
Latent tuberculosis	82 (18.7)	26 (17.9)	38 (18.5)	18 (20.2)	0.138
Cerebrovascular disease	4 (0.9)	1 (0.7)	0 (0.0)	3 (3.4)	0.012
Chronic pulmonary disease	31 (7.1)	1 (0.7)	15 (7.3)	15 (16.9)	<0.001
Interstitial lung disease	28 (6.4)	7 (4.8)	2 (1.0)	19 (21.4)	<0.001
Peptic ulcer disease	7 (1.6)	0 (0.0)	5 (2.4)	2 (2.3)	0.131
Mild liver disease	12 (2.7)	7 (4.8)	3 (1.5)	2 (2.3)	0.187
Diabetes mellitus	31 (7.1)	15 (10.3)	11 (5.4)	5 (5.6)	0.168
Renal disease	5 (1.1)	3 (2.1)	0 (0.0)	2 (2.3)	0.046
Non-metastatic solid tumor	13 (3.0)	8 (5.5)	3 (1.5)	2 (2.3)	0.084
Lymphoma	1 (0.2)	0 (0.0)	0 (0.0)	1 (1.1)	0.203
Metastatic solid tumor	1 (0.2)	1 (0.7)	0 (0.0)	0 (0.0)	0.533
**Number of comorbidities**					
0	343 (78.1)	110 (75.9)	171 (83.4)	62 (69.7)	0.054
1	83 (18.9)	30 (20.7)	31 (15.1)	22 (24.7)	
≥2	13 (3.0)	5 (3.5)	3 (1.5)	5 (5.6)	
CCI score	0.3 ± 0.7	0.4 ± 0.9	0.2 ± 0.5	0.4 ± 0.7	0.017
**RA disease activity**					
DAS28-ESR	6.5 ± 0.7	6.5 ± 0.6	6.4 ± 0.7	6.6 ± 0.8	0.026
Physician’s global assessment	73.6 ± 14.6	79.8 ± 10.8	69.4 ± 15.8	73.4 ± 13.8	<0.001
**Patient-reported outcomes**					
HAQ-DI	1.7 ± 0.8	1.3 ± 0.7	1.9 ± 0.7	1.9 ± 0.8	<0.001
EQ-5D	0.6 ± 0.2	0.7 ± 0.2	0.6 ± 0.2	0.6 ± 0.2	0.020
**Laboratory test**					
RF or ACPA positivity	407 (92.7)	129 (89.0)	191 (93.2)	87 (97.8)	0.040
ESR, mm/hr	56.0 ± 27 4	54.0 ± 26.9	54.7 ± 28.0	62.1 ± 26.3	0.015
CRP, mg/dL	2.4 ± 2.5	2.3 ± 2.5	2.1 ± 1.9	3.3 ± 3.5	0.050
**Ever used csDMARDs**					
Methotrexate	430 (98.0)	144 (99.3)	202 (98.5)	84 (94.4)	0.032
Leflunomide	256 (58.3)	95 (65.5)	119 (58.1)	42 (47.2)	0.022
Hydroxychloroquine	294 (67.0)	88 (60.7)	143 (69.8)	63 (70.8)	0.143
Sulfasalazine	258 (58.8)	75 (51.7)	133 (64.9)	50 (56.2)	0.041
Tacrolimus	152 (34.6)	64 (44.1)	55 (26.8)	33 (37.1)	0.003
Bucillamine	59 (13.4)	13 (9.0)	32 (15.6)	14 (15.7)	0.155
Azathioprine	8 (1.8)	1 (0.7)	3 (1.5)	4 (4.5)	0.129
Number of csDMARDs ever used	3.4 ± 1.1	3.3 ± 1.1	3.4 ± 1.1	3.3 ± 1.1	0.753
**Concomitant medications**					
Methotrexate	394 (89.8)	126 (86.9)	196 (95.6)	72 (80.9)	<0.001
Methotrexate dose, mg/week	12.8 ± 3.0	12.5 ± 2.7	13.2 ± 2.8	12.0 ± 3.6	0.016
NSAIDs	352 (80.2)	112 (77.2)	171 (83.4)	69 (77.5)	0.276
Oral glucocorticoid	348 (79.3)	111 (76.6)	160 (78.1)	77 (86.5)	0.159
Glucocorticoid dose, mg/day	5.5 ± 2.9	5.2 ± 2.9	5.0 ± 2.3	6.8 ± 3.6	0.001

Values are presented as means ± standard deviations or numbers (%).

RA, rheumatoid arthritis; JAKi, Janus kinase inhibitor; TNF, tumor necrosis factor; CCI, Charlson comorbidity index; DAS, disease activity score; ESR, erythrocyte sedimentation rate; HAQ-DI, health assessment questionnaire-disability index; EQ-5D, Euro-Qol 5-dimension; RF, rheumatoid factor; ACPA, anti-citrullinated protein antibody; CRP, C-reactive protein; csDMARDs, conventional synthetic disease-modifying antirheumatic drugs; NSAIDs, non-steroidal anti-inflammatory drugs.

The frequency of each drug selected to start is presented in S1 Fig. Adalimumab was the most frequently started medication (25.1%) followed by tofacitinib (19.4%) and abatacept (16.4%).

### Factors associated with selecting JAKis

In comparisons between JAKis and bDMARDs using binary logistic regression, patients with longer disease duration (OR 1.04, 95% confidence interval (CI) 1.00–1.07) and higher disease activity by PGA (OR 1.80, CI 1.45–2.23) were more likely to start JAKis than bDMARDs (Table 2). Moreover, patients who have ever used tacrolimus were more likely to start JAKis (OR 1.88, CI 1.15–3.08). On the other hand, patients with chronic pulmonary diseases (OR 0.06, CI 0.01–0.50) and higher HAQ-DI score (OR 0.31, CI 0.17–0.55) started bDMARDs rather than JAKis.

**Table 2:** Factors for starting JAKi compared with bDMARDs using binary logistic regression (n = 439).

Variables	Univariable analysis		Multivariable analysis*	
	Unadjusted OR (95% CI)	*P*	Adjusted OR (95% CI)	*P*
Sex, female	0.56 (0.31–1.01)	0.054	0.54 (0.25–1.19)	0.126
Age, years	1.02 (1.00–1.03)	0.025	1.01 (0.99–1.03)	0.242
Duration of RA, years	1.04 (1.02–1.07)	0.001	1.04 (1.00–1.07)	0.034
Body mass index, kg/m^2^ (n = 437)	1.01 (0.96–1.07)	0.732	1.02 (0.95–1.09)	0.548
**Comorbidities**				
Chronic pulmonary diseases	0.06 (0.01–0.45)	0.006	0.06 (0.01–0.50)	0.009
Interstitial lung disease	0.66 (0.27–1.59)	0.354	0.51 (0.16–1.65)	0.263
Diabetes mellitus	2.31 (1.13–4.71)	0.022	2.48 (1.00–6.16)	0.051
Mild liver diseases	2.93 (0.91–9.40)	0.071		
CCI score	1.27 (0.96–1.68)	0.091		
**RA disease activity**				
DAS28-ESR	1.26 (0.93–1.69)	0.132	1.18 (0.81–1.73)	0.390
Physician’s global assessment	1.76 (1.46–2.11)	<0.001	1.80 (1.45–2.23)	<0.001
**Patient-reported outcomes**				
HAQ-DI ≥ 1.0	0.42 (0.26–0.67)	<0.001	0.31 (0.17–0.55)	<0.001
**Laboratory test**				
RF or ACPA positivity	0.46 (0.23–0.96)	0.038	0.62 (0.26–1.48)	0.281
ESR, mm/hr	1.00 (0.99–1.00)	0.297		
CRP, mg/dL	0.98 (0.91–1.07)	0.656		
**Medication**				
Methotrexate	4.02 (0.50–32.47)	0.191	4.91 (0.46–52.00)	0.186
Leflunomide	1.57 (1.04–2.37)	0.032	1.27 (0.77–2.10)	0.348
Hydroxychloroquine	0.66 (0.44–1.00)	0.050	0.80 (0.48–1.33)	0.396
Sulfasalazine	0.65 (0.44–0.97)	0.036	0.78 (0.48–1.27)	0.313
Tacrolimus	1.85 (1.23–2.79)	0.003	1.88 (1.15–3.08)	0.012
NSAIDs	0.76 (0.47–1.24)	0.279		
Oral glucocorticoid	0.79 (0.49–1.27)	0.324	0.89 (0.51–1.55)	0.668

JAKi, Janus kinase inhibitor; bDMARDs, biological disease-modifying antirheumatic drugs; OR, odds ratio; CI, confidence interval; RA, rheumatoid arthritis; CCI, Charlson comorbidity index; DAS, disease activity score; ESR, erythrocyte sedimentation rate; HAQ-DI, health assessment questionnaire-disability index; RF, rheumatoid factor; ACPA, anti-citrullinated protein antibody; CRP, C-reactive protein; NSAIDs, non-steroidal anti-inflammatory drugs.

* The multivariable logistic regression analysis included 437 patients due to missing data for body mass index.

In multinomial logistic regression analysis, the JAKi group was less likely to have chronic pulmonary disease compared with the TNF inhibitor group (OR 0.07, CI 0.01–0.56) or the non-TNF inhibitor group (OR 0.06, CI 0.01–0.50) (Table 3). The PGA of disease activity was higher in the JAKi group than the TNF inhibitor group (OR 1.90, CI 1.51–2.38) or the non-TNF inhibitor group (OR 1.65, CI 1.28–2.14). However, JAKi users showed lower HAQ-DI than TNF inhibitor users (OR 0.27, CI 0.14–0.51) or non-TNF inhibitor users (OR 0.40, CI 0.18–0.90). In terms of age, JAKi users were older than TNF inhibitor users (OR 1.03, CI 1.01–1.05) but younger than non-TNF inhibitor users (OR 0.97, CI 0.95–1.00). The disease duration of RA was longer in JAKi users than non-TNF inhibitor users (OR 1.05, CI 1.00–1.09) but the difference was marginal when compared with TNF inhibitor users (OR 1.03, CI 0.99–1.07). In addition, patients with RA who had used tacrolimus were more likely to start JAKis than TNF inhibitors (OR 2.05, CI 1.20–3.51). Patients with ILD were more likely to receive non-TNF inhibitors than JAKis (OR 0.21, CI 0.06–0.72), but the reverse was true for patients with diabetes mellitus (OR 5.00, CI 1.24–20.22). The use of glucocorticoids was not a factor associated with choosing the type of targeted therapy.

**Table 3:** Factors for starting JAKi compared with TNF inhibitor or non-TNF inhibitor using multinomial logistic regression (n = 439).

Variables	JAKi vs. TNF inhibitor				JAKi vs. Non-TNF inhibitor			
	Univariable analysis		Multivariable analysis*		Univariable analysis		Multivariable analysis*	
	Unadjusted OR (95% CI)	*P*	Adjusted OR (95% CI)	*P*	Unadjusted OR (95% CI)	*P*	Adjusted OR (95% CI)	*P*
Sex, female	0.51 (0.26–0.99)	0.045	0.66 (0.28–1.53)	0.331	0.67 (0.30–1.49)	0.326	0.36 (0.12–1.10)	0.072
Age, years	1.03 (1.02–1.05)	<0.001	1.03 (1.01–1.05)	0.012	0.98 (0.96–1.00)	0.038	0.97 (0.95–1.00)	0.049
Duration of RA, years	1.05 (1.02–1.08)	0.001	1.03 (0.99–1.07)	0.106	1.03 (1.00–1.07)	0.072	1.05 (1.00–1.09)	0.035
Body mass index, kg/m^2^	1.01 (0.95–1.08)	0.662	1.03 (0.95–1.10)	0.498	1.00 (0.93–1.08)	0.967	1.03 (0.94–1.13)	0.544
**Comorbidities**								
Chronic pulmonary diseases	0.09 (0.01–0.67)	0.019	0.07 (0.01–0.56)	0.013	0.03 (0.00–0.26)	0.001	0.06 (0.01–0.50)	0.009
Interstitial lung disease	5.15 (1.05–25.17)	0.043	4.03 (0.63–25.69)	0.140	0.19 (0.08–0.47)	<0.001	0.21 (0.06–0.72)	0.013
Diabetes mellitus	2.34 (1.06–5.16)	0.035	1.81 (0.67–4.92)	0.243	2.23 (0.79–6.27)	0.129	5.00 (1.24–20.22)	0.024
Mild liver diseases	3.42 (0.87–13.44)	0.079			2.21 (0.45–10.87)	0.331		
CCI score	1.62 (1.14–2.30)	0.008			0.94 (0.68–1.30)	0.708		
**RA disease activity**								
DAS28-ESR	1.41 (1.03–1.94)	0.035	1.19 (0.79–1.80)	0.398	0.96 (0.65–1.43)	0.844	1.19 (0.72–1.96)	0.489
Physician’s global assessment	1.86 (1.53–2.26)	<0.001	1.90 (1.51–2.38)	<0.001	1.55 (1.24–1.93)	<0.001	1.65 (1.28–2.14)	<0.001
**Patient-reported outcomes**								
HAQ-DI ≥ 1.0	0.41 (0.24–0.69)	0.001	0.27 (0.14–0.51)	<0.001	0.43 (0.22–0.84)	0.013	0.40 (0.18–0.90)	0.027
**Laboratory test**								
RF or ACPA positivity	0.59 (0.28–1.25)	0.170	0.80 (0.32–1.97)	0.626	0.19 (0.04–0.83)	0.027	0.25 (0.05–1.31)	0.102
ESR, mm/hr	1.00 (0.99–1.01)	0.824			0.99 (0.98–1.00)	0.031		
CRP, mg/dL	1.05 (0.96–1.16)	0.286			0.89 (0.81–0.98)	0.014		
**Medication**								
Methotrexate	2.14 (0.22–20.77)	0.512	6.40 (0.42–97.82)	0.182	8.57 (0.99–74.61)	0.052	7.39 (0.51–106.92)	0.142
Leflunomide	0.73 (0.47–1.13)	0.158	1.19 (0.69–2.03)	0.537	0.47 (0.27–0.81)	0.006	1.44 (0.75–2.75)	0.274
Hydroxychloroquine	1.49 (0.96–2.34)	0.078	0.81 (0.47–1.39)	0.444	1.57 (0.89–2.76)	0.118	0.80 (0.41–1.54)	0.498
Sulfasalazine	1.72 (1.12–2.66)	0.014	0.68 (0.40–1.15)	0.146	1.20 (0.70–2.03)	0.507	1.05 (0.56–1.99)	0.873
Tacrolimus	2.16 (1.37–3.38)	0.001	2.05 (1.20–3.51)	0.008	1.34 (0.78–2.30)	0.288	1.70 (0.89–3.24)	0.108
NSAIDs	0.68 (0.40–1.15)	0.150			0.98 (0.52–1.85)	0.959		
Oral glucocorticoid	0.92 (0.55–1.52)	0.741	1.05 (0.58–1.90)	0.871	0.51 (0.25–1.05)	0.066	0.59 (0.27–1.32)	0.202

JAKi, Janus kinase inhibitor; TNF, tumor necrosis factor; OR, odds ratio; CI, confidence interval; RA, rheumatoid arthritis; CCI, Charlson comorbidity index; DAS, disease activity score; ESR, erythrocyte sedimentation rate; HAQ-DI, health assessment questionnaire-disability index; RF, rheumatoid factor; ACPA, anti-citrullinated protein antibody; CRP, C-reactive protein; NSAIDs, non-steroidal anti-inflammatory drugs.

* Two patients in the JAKi group were excluded in the multivariable logistic regression analysis due to missing data for body mass index.

## Discussion

In this study, we identified factors determining whether to start JAKi in csDMARD-IR patients with RA. About one-third of the patients in our study received JAKis, indicating that JAKi use kept up with TNF inhibitor and non-TNF inhibitor use. In a comparison between JAKis and bDMARDs, longer disease duration, higher disease activity assessed by PGA, and previous use of tacrolimus were factors favoring the selection of JAKis over bDMARDs, while the presence of chronic pulmonary diseases and higher functional disability were factors for prescribing bDMARDs rather than JAKis. However, we identified additional trends in the choice of targeted therapy when we divided the bDMARDs group into two groups, i.e., a TNF inhibitor and a non-TNF inhibitor group. In a comparison with the TNF inhibitor group, older age was an additional factor favoring prescription of JAKis. In a comparison with the non-TNF inhibitor group, younger age, diabetes mellitus and absence of ILD were factors influencing the prescription of JAKis.

There were common factors affecting the decision to start JAKis, indicating the same direction when compared with both TNF inhibitor and non-TNF inhibitor groups: higher disease activity assessed by physician, low HAQ-DI, and the absence of chronic pulmonary diseases. Higher disease activity assessed by physician is a physician-related factor, while low HAQ-DI and being less likely to have chronic pulmonary diseases are considered patient-related factors. However, in real-world practice, HAQ-DI and chronic pulmonary diseases could also influence physician decisions rather than patient decisions. In other words, physicians preferred JAKis when RA disease activity was high but did not prefer to prescribe JAKis for patients who had high functional disability or chronic pulmonary diseases. This suggests that JAKi was prescribed to a very unique population with higher disease activity and low HAQ-DI, since HAQ-DI is known to have positive correlations with disease activity such as DAS28-ESR [29].

In terms of disease activity of RA, it was interesting that objective indicators such as ESR, CRP, and DAS28-ESR were not important factors for selecting JAKis instead of TNF inhibitor or non-TNF inhibitor. Only PGA of disease activity, a subjective indicator assessed by physicians, was a significant factor in this study. The calculation for DAS28-ESR includes the patient’s global assessment, and discordance between DAS28-ESR and PGA has been reported by previous studies, suggesting discrepancies between patient and physician judgements of disease activity [30]. The reasons that physicians prescribed JAKis for patients with high PGA scores might be the novelty of JAKi and the rapid onset of pharmacologic effects [31]. Further study is necessary to determine the reasons underlying therapy choice and if this information is shared with patients.

There were fewer patients with chronic pulmonary diseases in the JAKi group, which means that those with chronic pulmonary diseases tended to receive a TNF inhibitor or non-TNF inhibitor. Considering that comorbidities such as COPD and asthma were included as chronic pulmonary diseases, the main reason might be insufficient data on the pulmonary safety of JAKis in clinical trials [21,32]. Non-TNF inhibitor were more frequently used by patients with ILD than JAKis. There have been studies suggesting that non-TNF inhibitors slow the worsening of ILD more effectively than TNF inhibitors [33,34], and this may have been the reason for selecting non-TNF inhibitors in patients with ILD in the present study.

Patients with diabetes mellitus were more likely to receive JAKis than non-TNF inhibitors. This finding is contrary to a previous report that abatacept users had a lower incidence of diabetes mellitus than TNF inhibitor users [35]. Though drug use in patients with diabetes mellitus was not investigated in detail, patients who used subcutaneous insulin or were currently using it may have preferred JAKis because they were orally administered. This finding needs confirmation.

Tacrolimus use was also revealed to affect JAKi initiation when compared with TNF inhibitor. Tacrolimus is not commonly used first, compared to other csDMARDs such as MTX, leflunomide, hydroxychloroquine, and sulfasalazine. In addition, tacrolimus is mainly used for RA treatment in the Asia-Pacific region [36], while it is not included as a csDMARD in the guidelines published by the EULAR or ACR [9,10]. This finding suggests two main points. First, the initiation of targeted therapy for Korean patients with RA seems to have been delayed. As shown in Table 1, tacrolimus has ever been used in 34.6% of total patients who started targeted therapy, and even more frequently, in 44.1% of JAKi starters. In other words, this means that patients starting targeted therapy had IR to multiple csDMARDs. This correlates with the result that the average duration of RA at the initiation of targeted therapy was as long as 7.6 years. This may be due to the demanding criteria for providing reimbursement for targeted therapy required by the national healthcare insurance system in Korea and patient reluctance to start new drugs. Second, the fact that many patients in the JAKi group have used tacrolimus could indicate patient preference for the oral route of administration. In the same vein, the longer duration of RA in the JAKi group suggests that they preferred csDMARDs and delayed targeted therapy. Patients reluctant to start injections or infusions might delay targeted therapy even until they used tacrolimus.

The age of patients showed different directions in the JAKi group when compared with TNF inhibitor or non-TNF inhibitor group. Patients who started JAKis were older than TNF inhibitor starters and younger than non-TNF inhibitor starters. This also explains why there were significantly fewer MTX users in the non-TNF inhibitor group.

This study was meaningful in several respects. First, we identified several factors for selecting the type of targeted therapy in a real-world setting. According to the current guidelines for RA treatment published by EULAR and ACR, JAKis, TNF inhibitors, and non-TNF inhibitors are recommended for csDMARD-IR patients equally by shared decision-making process [9,10]. In the real world, however, patients have little choice but to rely on their physician decisions due to limitations of knowledge. Nevertheless, we indirectly demonstrated that selection of the type of targeted therapy could depend on the formulation of the drug, regardless of its efficacy and adverse effects. This should be considered an important bias when analyzing comparative efficacy between medications with different formula. Second, we were able to raise questions about the delay of targeted therapy for RA patients in Korea through the results in this study. Further study using real-world data is necessary to provide guidance to improve policies such as reimbursement criteria.

There are limitations of this study because we could not include several factors. First, socioeconomic factors of patient could not be analyzed in this study. Patient-related factors such as education, income, and insurance type were not included for the cohorts from which we extracted data. The intellectual and economic statuses of patients could influence the choice of the drug. However, the costs of each targeted therapy were similar, so cost should have no significant impact. Second, this study was a single-center study and the results may not be generalizable. As an academic referral hospital, there could be selection bias because patients were more likely to have higher disease activity. However, our hospital houses the largest rheumatology center in Korea, with numeral rheumatologists and patients from all over the country. Further studies with large populations are necessary to clarify our findings about factors for selecting the type of targeted therapy, and for assessing the effectiveness of shared decision-making by patient empowerment. In addition, the comparative effectiveness and safety of targeted therapies in patients preferring particular therapy type should be studied.

In conclusion, age, absence of pulmonary comorbidities, previous history of taking tacrolimus, and high disease activity assessed by PGA were factors influencing selecting JAKis rather than bDMARDs when RA patients started targeted therapy. To elucidate the factors underlying selection of type of targeted therapy, further studies with large sample sizes that include socioeconomic status of patients and assess the effectiveness of shared decision-making processes would be informative.

## Supporting information

S1 Fig Number of patients by type of targeted therapy. JAKi, Janus kinase inhibitor, TNF, tumor necrosis factor.
(TIF)

## References

[1] Curtis JR, Singh JA. Use of biologics in rheumatoid arthritis: current and emerging paradigms of care. Clin Ther. 2011;33(6):679–707. doi: 10.1016/j.clinthera.2011.05.044 21704234 PMC3707489

[2] Rein P, Mueller RB. Treatment with Biologicals in Rheumatoid Arthritis: An Overview. Rheumatol Ther. 2017;4(2):247–61. doi: 10.1007/s40744-017-0073-3 28831712 PMC5696285

[3] Burmester GR, Pope JE. Novel treatment strategies in rheumatoid arthritis. The Lancet. 2017;389(10086):2338–48. doi: 10.1016/S0140-6736(17)31491-5 28612748

[4] Burmester GR, Panaccione R, Gordon KB, McIlraith MJ, Lacerda AP. Adalimumab: long-term safety in 23 458 patients from global clinical trials in rheumatoid arthritis, juvenile idiopathic arthritis, ankylosing spondylitis, psoriatic arthritis, psoriasis and Crohn’s disease. Ann Rheum Dis. 2013;72(4):517–24. doi: 10.1136/annrheumdis-2011-201244 22562972 PMC3595151

[5] Atzeni F, Sarzi-Puttini P, Mutti A, Bugatti S, Cavagna L, Caporali R. Long-term safety of abatacept in patients with rheumatoid arthritis. Autoimmun Rev. 2013;12(12):1115–7. doi: 10.1016/j.autrev.2013.06.011 23800448

[6] Cohen SB, Tanaka Y, Mariette X, Curtis JR, Lee EB, Nash P, et al. Long-term safety of tofacitinib up to 9.5 years: a comprehensive integrated analysis of the rheumatoid arthritis clinical development programme. RMD Open. 2020;6(3). 10.1136/rmdopen-2020-001395 PMC7722371 33127856

[7] Fleischmann R, Takeuchi T, Schiff M, Schlichting D, Xie L, Issa M, et al. Efficacy and Safety of Long-Term Baricitinib With and Without Methotrexate for the Treatment of Rheumatoid Arthritis: Experience With Baricitinib Monotherapy Continuation or After Switching From Methotrexate Monotherapy or Baricitinib Plus Methotrexate. Arthritis Care Res (Hoboken). 2020;72(8):1112–21. doi: 10.1002/acr.24007 31233281

[8] Kerschbaumer A, Sepriano A, Smolen JS, van der Heijde D, Dougados M, van Vollenhoven R, et al. Efficacy of pharmacological treatment in rheumatoid arthritis: a systematic literature research informing the 2019 update of the EULAR recommendations for management of rheumatoid arthritis. Ann Rheum Dis. 2020;79(6):744–59. doi: 10.1136/annrheumdis-2019-216656 32033937 PMC7286044

[9] Smolen JS, Landewe RBM, Bijlsma JWJ, Burmester GR, Dougados M, Kerschbaumer A, et al. EULAR recommendations for the management of rheumatoid arthritis with synthetic and biological disease-modifying antirheumatic drugs: 2019 update. Ann Rheum Dis. 2020;79:685–99. doi: 10.1136/annrheumdis-2019-216655 31969328

[10] Fraenkel L, Bathon JM, England BR, St Clair EW, Arayssi T, Carandang K, et al. 2021 American College of Rheumatology Guideline for the Treatment of Rheumatoid Arthritis. Arthritis Care Res (Hoboken). 2021;73(7):924–39. doi: 10.1002/acr.24596 34101387 PMC9273041

[11] Park EJ, Kim H, Jung SM, Sung YK, Baek HJ, Lee J. The use of biological disease-modifying antirheumatic drugs for inflammatory arthritis in Korea: results of a Korean Expert Consensus. Korean J Intern Med. 2020;35(1):41–59. doi: 10.3904/kjim.2019.411 31935319 PMC6960050

[12] Sepriano A, Kerschbaumer A, Smolen JS, van der Heijde D, Dougados M, van Vollenhoven R, et al. Safety of synthetic and biological DMARDs: a systematic literature review informing the 2019 update of the EULAR recommendations for the management of rheumatoid arthritis. Ann Rheum Dis. 2020;79(6):760–70. doi: 10.1136/annrheumdis-2019-216653 32033941

[13] Lee YH, Bae SC. Comparative efficacy and safety of tocilizumab, rituximab, abatacept and tofacitinib in patients with active rheumatoid arthritis that inadequately responds to tumor necrosis factor inhibitors: a Bayesian network meta-analysis of randomized controlled trials. Int J Rheum Dis. 2016;19(11):1103–11. doi: 10.1111/1756-185X.12822 26692536

[14] Huoponen S, Aaltonen KJ, Viikinkoski J, Rutanen J, Relas H, Taimen K, et al. Cost-effectiveness of abatacept, tocilizumab and TNF-inhibitors compared with rituximab as second-line biologic drug in rheumatoid arthritis. PLoS One. 2019;14(7):e0220142. doi: 10.1371/journal.pone.0220142 31339961 PMC6656352

[15] Simon TA, Boers M, Hochberg M, Baker N, Skovron ML, Ray N, et al. Comparative risk of malignancies and infections in patients with rheumatoid arthritis initiating abatacept versus other biologics: a multi-database real-world study. Arthritis Res Ther. 2019;21(1):228. doi: 10.1186/s13075-019-1992-x 31703717 PMC6839238

[16] Chen SK, Liao KP, Liu J, Kim SC. Risk of Hospitalized Infection and Initiation of Abatacept Versus Tumor Necrosis Factor Inhibitors Among Patients With Rheumatoid Arthritis: A Propensity Score-Matched Cohort Study. Arthritis Care Res (Hoboken). 2020;72(1):9–17. 10.1002/acr.23824 PMC6586544 30570833

[17] Suissa S, Hudson M, Dell’Aniello S, Shen S, Simon TA, Ernst P. Comparative safety of abatacept in rheumatoid arthritis with COPD: A real-world population-based observational study. Semin Arthritis Rheum. 2019;49(3):366–72. doi: 10.1016/j.semarthrit.2019.03.007 30979397

[18] Cassone G, Manfredi A, Atzeni F, Venerito V, Vacchi C, Picerno V, et al. Safety of Abatacept in Italian Patients with Rheumatoid Arthritis and Interstitial Lung Disease: A Multicenter Retrospective Study. J Clin Med. 2020;9(1):277. doi: 10.3390/jcm9010277 31963908 PMC7019755

[19] Jang SH, Ju JH. Janus kinase inhibitors for the treatment of rheumatoid arthritis. J Korean Med Assoc. 2021;64(2):105–8.

[20] Hsiao B, Fraenkel L. Patient preferences for rheumatoid arthritis treatment. Curr Opin Rheumatol. 2019;31(3):256–63. doi: 10.1097/BOR.0000000000000591 30747733 PMC6438722

[21] Harigai M. Growing evidence of the safety of JAK inhibitors in patients with rheumatoid arthritis. Rheumatology (Oxford). 2019;58(Suppl 1):i34–i42. doi: 10.1093/rheumatology/key287 30806708 PMC6390880

[22] Verden A, Dimbil M, Kyle R, Overstreet B, Hoffman KB. Analysis of spontaneous postmarket case reports submitted to the FDA regarding thromboembolic adverse events and JAK inhibitors. Drug Saf 2018;41(4):357–61. doi: 10.1007/s40264-017-0622-2 29196988

[23] Kalkan A, Husberg M, Hallert E, Roback K, Thyberg I, Skogh T, et al. Physician Preferences and Variations in Prescription of Biologic Drugs for Rheumatoid Arthritis: A Register-Based Study of 4,010 Patients in Sweden. Arthritis Care Res (Hoboken). 2015;67(12):1679–85. doi: 10.1002/acr.22640 26097219

[24] Louder AM, Singh A, Saverno K, Cappelleri JC, Aten AJ, Koenig AS, et al. Patient preferences regarding rheumatoid arthritis therapies: a conjoint analysis. Am Health Drug Benefits 2016;9(2):84–93. 27182427 PMC4856233

[25] Emadi SA, Hammoudeh M, Mounir M, Mueller RB, Wells AF, Sarakbi HA. An assessment of the current treatment landscape for rheumatology patients in Qatar: Recognising unmet needs and moving towards solutions. J Int Med Res 2017;45(2):733–43. doi: 10.1177/0300060516686872 28415924 PMC5536653

[26] Davari M, Khorasani E, Tigabu BM. Factors influencing prescribing decisions of physicians: A Review. Ethiop J Health Sci 2018;28(6):795–804. doi: 10.4314/ejhs.v28i6.15 30607097 PMC6308758

[27] Health Insurance Review and Assessment Service. List of reimbursable drugs. 2015 [Cited 2022 Oct 3]. Available from: https://www.hira.or.kr/bbsDummy.do?pgmid=HIRAA030014050000&brdScnBltNo=4&brdBltNo=1550&pageIndex=11&pageIndex2=11#none.

[28] Cho SK, Kim D, Jun JB, Bae SC, Sung YK. Factors influencing quality of life (QOL) for Korean patients with rheumatoid arthritis (RA). Rheumatol Int 2013;33(1):93–102. doi: 10.1007/s00296-011-2352-6 22218643

[29] Kumar BS, Suneetha P, Mohan A, Kumar DP, Sarma KVS. Comparison of Disease Activity Score in 28 joints with ESR (DAS28), Clinical Disease Activity Index (CDAI), Health Assessment Questionnaire Disability Index (HAQ-DI) & Routine Assessment of Patient Index Data with 3 measures (RAPID3) for assessing disease activity in patients with rheumatoid arthritis at initial presentation. Indian J Med Res. 2017;146(Supplement):S57–S62. 29578196 10.4103/ijmr.IJMR_701_15 PMC5890597

[30] Desthieux C, Hermet A, Granger B, Fautrel B, Gossec L. Patient-physician discordance in global assessment in rheumatoid arthritis: A systematic literature review with meta-analysis. Arthritis Care Res (Hoboken) 2016;68(12):1767–73. doi: 10.1002/acr.22902 27059693

[31] Angelini J, Talotta R, Roncato R, Fornasier G, Barbiero G, Dal Cin L, et al. JAK-inhibitors for the treatment of rheumatoid arthritis: A focus on the present and an outlook on the future. Biomolecules 2020;10(7):1002. doi: 10.3390/biom10071002 32635659 PMC7408575

[32] Saldarriaga-Rivera LM, López-Villegas VJ. Janus kinase inhibitors as a therapeutic option in rheumatoid arthritis and associated interstitial lung disease. Report of four cases. Revista Colombiana de Reumatología (English Edition) 2019;26(2):137–9.

[33] Mena-Vazquez N, Godoy-Navarrete FJ, Manrique-Arija S, Aguilar-Hurtado MC, Romero-Barco CM, Urena-Garnica I, et al. Non-anti-TNF biologic agents are associated with slower worsening of interstitial lung disease secondary to rheumatoid arthritis. Clin Rheumatol 2021;40(1):133–42. doi: 10.1007/s10067-020-05227-9 32557255

[34] Vicente-Rabaneda EF, Atienza-Mateo B, Blanco R, Cavagna L, Ancochea J, Castaneda S, et al. Efficacy and safety of abatacept in interstitial lung disease of rheumatoid arthritis: A systematic literature review. Autoimmun Rev 2021;20(6):102830. doi: 10.1016/j.autrev.2021.102830 33887489

[35] Desai RJ, Dejene S, Jin Y, Liu J, Kim SC. Comparative Risk of Diabetes Mellitus in Patients With Rheumatoid Arthritis Treated With Biologic or Targeted Synthetic Disease-Modifying Drugs: A Cohort Study. ACR Open Rheumatol 2020;2(4):222–31. doi: 10.1002/acr2.11124 32267094 PMC7164631

[36] Lau CS, Chia F, Dans L, Harrison A, Hsieh TY, Jain R, et al. 2018 update of the APLAR recommendations for treatment of rheumatoid arthritis. Int J Rheum Dis 2019;22(3):357–75. doi: 10.1111/1756-185X.13513 30809944

