# Supplementary figures and images for: Factors associated with selection of targeted therapy in patients with rheumatoid arthritis
Source: PLoS One. 2023 Jan 10;18(1):e0280234. doi: 10.1371/journal.pone.0280234 (PMC9831325; doi:10.1371/journal.pone.0280234)

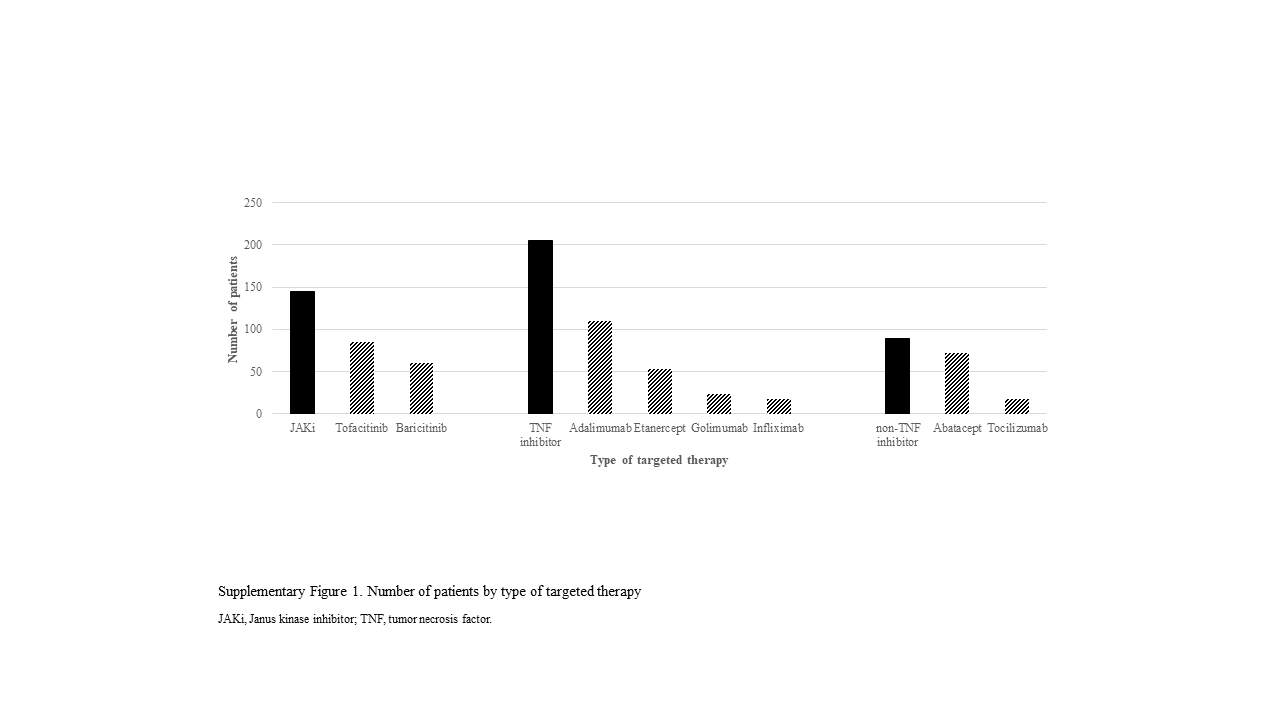

Supplement: S1 Fig — JAKi, Janus kinase inhibitor, TNF, tumor necrosis factor. (TIF) [file pone.0280234.s001.tif]
